# Attitudes of Australasian Clinicians and Laboratory Staff to Changing Fungal Nomenclature: Has Mycological Correctness Really Gone Mad?

**DOI:** 10.1128/spectrum.02377-21

**Published:** 2022-02-09

**Authors:** Sarah E. Kidd, Catriona L. Halliday, Elizabeth Haremza, Dianne J. Gardam, Sharon C. A. Chen, Juliet A. Elvy

**Affiliations:** a National Mycology Reference Centre, Microbiology and Infectious Diseases, South Australia Pathology, Adelaide, South Australia, Australia; b Royal College of Pathologists of Australasia Quality Assurance Programs (RCPAQAP), Microbiology Section, St. Leonards, Sydney, New South Wales, Australia; c Centre for Infectious Diseases and Microbiology Laboratory Services, ICPMR, New South Wales Health Pathology, Westmead Hospital, Westmead, New South Wales, Australia; d PathWest Laboratory Medicine WA, Department of Microbiology, Fiona Stanley Hospital, Murdoch, Western Australia, Australia; e Department of Microbiology, Wellington Southern Community Laboratories, Wellington Regional Hospital, Newtown, Wellington, New Zealand; MRC Centre Medical Mycology at the University of Exeter

**Keywords:** mycology, nomenclature, *Candida*, *Pichia kudriavzeveii*, *Nakaseomyces glabrata*

## Abstract

Fungal nomenclature changes have been a regular occurrence in recent years, eliciting heated debate on whether such changes will confuse clinicians and harm patients. We conducted surveys of Australasian laboratory staff and clinicians to assess attitudes, practices, and concerns regarding nomenclatural change. The majority of respondents to both surveys were aware of fungal nomenclatural changes (93.5% laboratories, 79.7% clinicians); 72.8% of laboratories had already implemented nomenclature changes, and 68.7% of clinicians recalled receiving at least one laboratory report utilizing updated fungal nomenclature. The vast majority of clinicians (94%) both within and outside of infection specialties supported laboratories reporting updated species names with inclusion of the previous species name. The importance of including the previous name on reports was demonstrated by 73.3% of clinicians viewing “*Nakaseomyces glabrata* (formerly Candida glabrata)” as clinically significant, versus only 38.2% viewing “*Pichia kudriavzeveii*” as significant in the absence of its former name. When asked about reporting practices, 73.9% of laboratories would report a Candida krusei isolate as “*Pichia kudriavzeveii* (formerly Candida krusei),” with the rest reporting as “Candida krusei*”* (21.7%) or “*Pichia kudriavzeveii”* (1.1%) without further explanation. Laboratory concerns included clinicians being confused by reports, commonly used identification platforms continuing to use superseded species names, education of staff, and delays in updating species codes in laboratory information systems. Adopting fungal name changes appears to be well supported by laboratories and clinicians in Australia and New Zealand, and can be achieved safely and unambiguously provided the former name is included on reports.

**IMPORTANCE** Recent changes in fungal species names have been contentious, eliciting heated debate on social media. Despite available recommendations on adapting to the changes, concerns include clinicians dismissing pathogens as contaminants with patient harm as a result, and disruption of the literature. Such concerns are understandable, but are not supported by evidence and may represent a vocal minority. This survey of Australasian laboratories and clinicians assesses attitudes and practices relating to changes in fungal nomenclature and found that there is overwhelming support for adopting nomenclature changes.

## INTRODUCTION

Fungal nomenclature continues to undergo extensive change as molecular technologies replace conventional phenotypic methods of classification. Molecular techniques are better able to designate fungal species and more accurately define inter- and intra-species phylogenetic relationships, thereby correcting taxonomical errors arising from older conventional methods. Extensive genetic diversity revealed within some morphologically ascribed species has led to many new species being described (e.g., Aspergillus *lentulus* is among ∼50 species identified within the Aspergillus fumigatus complex); analysis of some genera has confirmed the species within them to be polyphyletic (e.g., *Candida* species, originally named as a group of white budding yeasts, are in many cases unrelated) warranting reclassification into appropriate genera. Furthermore, the long-held convention of fungal species having two or more valid names for their sexual states was abandoned in 2011 (1), and rationalization of existing names meant that some familiar names in common use were replaced by the less commonly used names (e.g., *Penicillium marneffei* was replaced by *Talaromyces marneffei*) (2).

Many name changes have been adopted with little controversy, while others have raised debate about whether the use of unfamiliar species names could lead to erroneous clinical decisions and even patient harm (3). Other concerns include the disruption of scientific literature, nucleotide databases, and antibiograms, as well as intensifying differences in laboratory reporting practices; while such concerns may be understandable, they are not currently supported by evidence.

In Australia and New Zealand, clinical microbiology laboratories receive annual updates about changes in fungal nomenclature via the mycology module of the Royal College of Pathologists Australasia Quality Assurance Program (RCPAQAP). Laboratories are provided with a list of fungal species that highlights changes and includes the previous (synonymized) names of fungi that may be dispatched throughout the program; it is the expectation that laboratories will make efforts to adopt updated nomenclature within 1 year. Anecdotally, some concerns about nomenclature changes have been raised with the RCPAQAP, but there has been no systematic attempt to document these issues. This prompted us to carry out the present survey-based study to assess the attitudes and concerns of microbiology laboratories and clinicians in Australia and New Zealand to changes in fungal nomenclature.

## RESULTS

Ninety-two laboratory representatives responded to Survey 1 and 217 clinicians responded to Survey 2. Most laboratory respondents were from government-funded laboratories (58/92, 63%), and the majority of clinicians were infectious diseases physicians or clinical microbiologists (157/217, 72.3%). Tables 1 and 2 summarize the characteristics and specialty areas of respondents.

**TABLE 1:** Summary of laboratories participating in the Survey 1

Laboratory characteristic	*N*	%
Laboratory type (*N* = 92)		
Private pathology laboratory	34	36.9
Government/public pathology laboratory (including reference laboratories)	58	63
Mycology capabilities of the laboratory (*N* = 92)		
Bacteriology laboratory with no specific mycology services (most fungal laboratory requests are referred)	5	5.4
Bacteriology laboratory with some limited mycology services: microscopy, culture, and basic identification	55	59.8
A specialized mycology service or reference laboratory: microscopy, culture, complex identifications, susceptibility testing	32	34.8
Respondent’s role in the laboratory (*N *= 92)		
Medical scientist working at the bench	38	41.3
Laboratory manager	12	13.0
Quality manager/officer	1	1.1
Clinical microbiologist/pathologist	41	44.6

**TABLE 2:** Areas of specialty for clinicians participating in Survey 2

Area of practice (*N* = 217)	*N*	%
Infectious diseases/microbiology, including pediatric infectious diseases	157	72.3
Hematology/oncology	25	11.5
Internal medicine	10	4.6
General practice	7	3.2
Immunology and allergy	7	3.2
Pediatrics/neonatology	5	2.3
Intensive care	3	1.4
Rheumatology	2	0.9
Veterinary medicine	1	0.5

### Survey 1: laboratory responses.

Of the 92 laboratory respondents, 86 (93.5%) were aware of recent fungal nomenclature changes and 71/92 (77.2%) indicated that it was appropriate to update reporting in accordance with accepted taxonomy; a majority indicated they had already implemented nomenclature changes (67/92, 72.8%). Four (4.3%) laboratories indicated that it was not appropriate to use updated nomenclature, 12 (13.0%) indicated it was appropriate to implement changes for some but not all species, and five (5.4%) were not sure. Most laboratories indicated that they apply updated nomenclature as communicated in the annually updated RCPAQAP List of QAP Fungi (available online to participants) (65/92, 70.7%). Sixty-eight laboratories (73.9%) indicated that an isolate identified as “Candida krusei” by matrix assisted laser desorption ionization-time of flight mass spectrometry (MALDI-ToF MS) or similar IVD platform would be reported as “*Pichia kudriavzevii* (formerly Candida krusei)” whereas 20 laboratories (21.7%) would report it as Candida krusei, and just one laboratory would report it as “*Pichia kudriavzevii*” without further comment. A majority of laboratories (69/92, 75%) indicated that they would refer to the RCPAQAP List of QAP Fungi to check the currently accepted nomenclature of a given species. Use of online databases such as MycoBank (mycobank.org) and Index Fungorum (indexfungorum.org), searching the scientific literature, or a “Google search” were also popular strategies.

Additional comments received from laboratories mainly centered around concerns about clinicians not recognizing the new fungal names, MALDI-ToF MS databases still using superseded names, delays in making the necessary coding changes in the laboratory information system, and ongoing requirements for education and training of staff.

### Survey 2: clinician responses.

**FIG 1:**
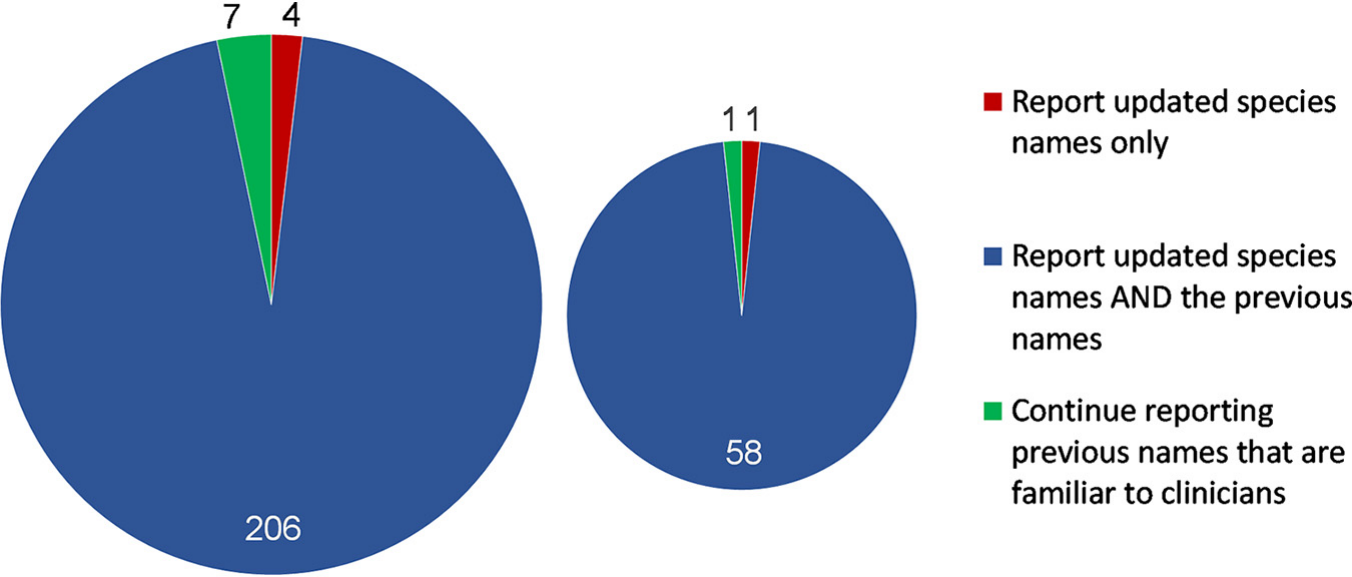
Responses of all clinicians (*n* = 217, left) and clinicians in non-infection specialties (*n* = 60, right) to the question “In your opinion how should laboratories report a significant fungal species that has undergone a name change?”

Of the 217 clinicians, 173 (79.7%) were aware of recent nomenclature changes and 149 (68.7%) had received at least one laboratory report utilizing an updated fungal species name, compared with 46 (21.2%) who had not, and 22 (10.1%) who were not sure. With respect to mycology laboratory reports, the overwhelming majority of clinicians indicated that the report should include both the updated species name as well as the previous species name (204/217, 94%), while a minority favored continuing to report the old familiar names (7/217, 3.2%), or reporting the updated species name alone (4/217, 1.8%) (Fig. 1). Importantly, 43 of the 44 (97.8%) clinicians who were not previously aware of fungal nomenclature changes were in favor of reporting updated species names along with the previous name. Because a majority of respondents identified as infectious diseases and/or clinical microbiologists, we assessed the responses of the non-infection specialty clinicians as a subgroup (*n* = 60). Among this subgroup, the vast majority were in favor of reporting both the updated and the previous species names together (58/60, 96.7%), while only one (1.7%) each were in favor of reporting the old name only, or the updated name alone (Fig. 1).

**FIG 2:**
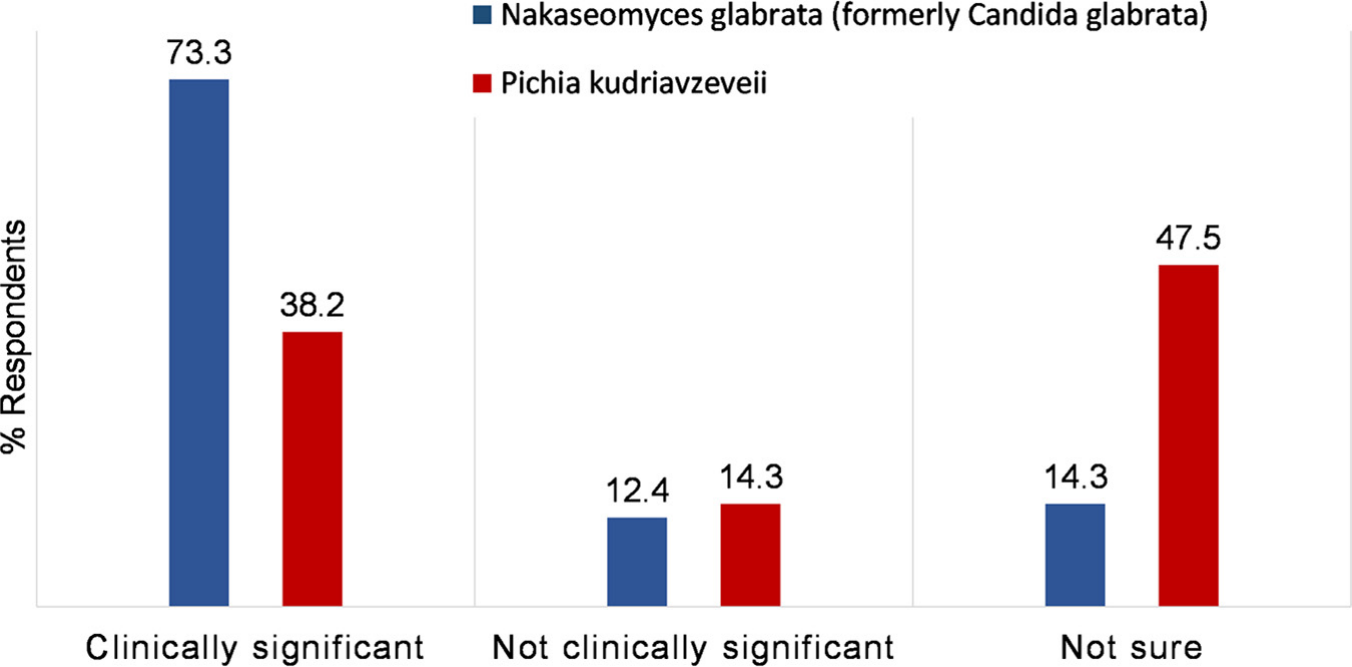
Clinicians’ (*n* = 217) responses to questions on the potential significance of fungal organisms with updated nomenclature, isolated from an abscess swab, reported with and without previous species names: *Nakaseomyces glabrata* (formerly Candida glabrata) versus *Pichia kudriavzeveii*.

To assess the value of including the previous species name on the report with updated names, clinicians were asked whether they would view as significant two organisms grown from an abscess, the first reported with updated and previous species name (“Growth of *Nakaseomyces glabrata* (formerly Candida glabrata)”), and the second reported with only the updated species name (“Growth of *Pichia kudriavzeveii”*). The majority (159/217, 73.3%) indicated that they would view “Growth of *Nakaseomyces glabrata* (formerly Candida glabrata)” as clinically significant compared with 38.2% for a report containing “Growth of *Pichia kudriavzeveii*” without the inclusion of the previous name (83/217, *P* < 0.00001) (Fig. 2). Similar responses were provided by clinicians in the non-infection specialty subgroup (63.3%, 38/60 considered “Growth of *Nakaseomyces glabrata* (formerly *Candida glabrata*)” significant versus 11.7%, 7/60 viewing ”*Pichia kudriavezeveii*” as significant, *P* < 0.00001).

The most common strategy identified by clinicians faced with an unfamiliar species names was to “Google search” (177/217, 81.6%), followed by a literature search (152/217, 70.0%), or discussion with the laboratory/pathologist (141/217, 64.9%). Four responses (1.8%) involved consideration of the clinical history and previous test results from the patient.

## DISCUSSION

Our survey of laboratory personnel and clinicians in Australia and New Zealand demonstrates a high level of awareness about changes to fungal species names and support for accommodating fungal name changes in clinical practice, provided that the old familiar names are included with the new name on the report. Those respondents who were unaware of nomenclature changes largely identified as either laboratories with limited or no mycology service, or clinicians outside of the infection-specialty areas.

Significantly more clinicians in our survey recognized the potential significance of *Nakaseomyces glabrata* because its previous name was provided, than for *Pichia kudriavzeveii* reported without its former name, Candida krusei. The same trend was observed within the subgroup of 60 clinicians from non-infection specialties. Therefore, the inclusion of previous species names on laboratory reports would appear to be critical in transitioning safely to the use of updated nomenclature. Laboratory staff require education and training to ensure this occurs consistently on reports to help embed the changes.

Freetext comments provided by clinicians in the survey that were in favor of reporting new fungal species along with their former names, included:

“Nomenclature changes are part of infectious diseases and we should strive to use the most up to date and appropriate names for organisms across the spectrum.”

“In the longer term, the update to fungal nomenclature is a good thing! In the shorter term, we need to ensure that clinical risk is minimized with good communication and education.”

“In general I think it is a good idea to use new species names. This is particularly true in the case of *Candida* species. Although clinicians may complain the new names are confusing they often provide a trigger that perhaps giving fluconazole is not always the answer.”

Of the small minority of clinicians (3.2%) that indicated a preference for continuing to report superseded species names despite nomenclature changes, some of the reasons or comments provided included:

“Mycological correctness has gone mad.”

“New names = contaminants. Ignore results.”

“New fungus names are impractical, clinicians have too many other important changes in medicine to be aware of. If it sounds funny or I cannot pronounce it, then it’s not important and it gets ignored.”

“It’s too hard to learn all the new fungus names when there are so many other significant changes in medicine to be aware of. Just keep things simple and stop unnecessary changes.”

Irrespective of personal feelings about “mycological correctness” and the comparative ease of species name pronunciation, nomenclature changes for microorganisms are not new and they occur for reasons that are scientifically sound. Nor are they unique to fungi (4–7). Some laboratory staff and clinicians may even recall the mid to late 1990s when Candida glabrata was still commonly referred to as *Torulopsis glabrata*; a nomenclature change for which a safe transition was evidently possible. This new reclassification of C. glabrata to the genus *Nakaseomyces* and C. krusei to *Pichia*, may seem unnecessary and confusing to some. But in fact, it places these species alongside their similarly fluconazole-resistant relatives, and when used consistently these genus names will become embedded in clinical decision making. Education and effective communication between clinical microbiology laboratories and clinicians, which is already integral to good laboratory practice, is critical to adapting to fungal nomenclature changes safely.

We observed a high level of awareness about changing fungal nomenclature; this may in part be due to the proactive actions taken by the RCPAQAP Microbiology Committee which provides an annual updated list of clinically important fungal species names. Obsolete species names are removed from the list available for mycology result submission and those participants reporting species names that have been communicated as obsolete for >1 year are scored with a minor discordance. Further commentary on nomenclature issues is provided in survey reports. By taking this approach, the QAP aims to promote consistent reporting among laboratories. And while this was not assessed in this survey, RCPAQAP data shows the rate of reporting obsolete species reduced from 43% to <1% in 2 years (3).

Concerns about the slowness of nomenclature updates in proprietary databases such as those for MALDI-ToF MS are certainly valid, and largely due to having to meet requirements of regulatory bodies. Certainly the Vitek MS Expanded V3.2 database (bioMérieux, Marcyl’Etoile, France) which has received 510(k) clearance from the U.S. Food and Drug Administration (FDA), uses some updated nomenclature (e.g., Purpureocillium lilacinum, *Lichthiemia coymbifera*, *Sarocladium kiliense*) but also obsolete nomenclature (e.g., *Candida* spp., *Scedosporium prolificans*). However, the recently released “MBT Compass Library Revision L” (Bruker Daltoniks, Bremen, Germany, November 2020) accommodates the reclassification of many yeasts.

A limitation of this study is that despite efforts to capture a wide range of clinician specialties, more than 70% of Survey 2 respondents were clinicians in infectious diseases and/or microbiology, and therefore largely represent the views of these specialties. No surgeons, dermatologists, respiratory physicians, or renal medicine physicians responded, which may reflect a lack of interest in taxonomic matters. Nevertheless, those that did respond expressed a similar view to the respondents from infection specialties.

In conclusion, our surveys support the view that fungal nomenclature changes can be implemented safely, if the previous and more clinically familiar species name is also included on the report; this approach is also recommended by others (5, 8–10). While some may believe that “mycological madness has gone mad,” our surveys suggest that most support the change.

## MATERIALS AND METHODS

Two electronic surveys comprising nine questions each were designed using the Microsoft Forms application, with all questions being compulsory (supplementary information). The surveys were designed by the authors with input from members of the Australian and New Zealand Mycoses Interest Group (ANZMIG), a special interest group of the Australasian Society of Infectious Diseases (ASID). Survey 1 was designed for microbiology laboratory staff and was distributed to Australia- and New Zealand-based participants in the RCPAQAP microbiology and mycology programs. Survey 2 was designed for clinicians, pathologists, fellows, and members of the following professional bodies: ASID, the Royal College of Pathologists of Australasia (RCPA), the Royal Australasian College of Physicians (RACP), the Royal Australian College of General Practitioners (RACGP), the Royal New Zealand College of General Practitioners (RNZCGP), the New Zealand Microbiology Network (NZMN), and the Australian and New Zealand Intensive Care Society (ANZICS). The intent was to include a wide range of medical specialties. Surveys were open to responses for 8 weeks between July 2021 and September 2021. Data for each survey was collated, and free-text responses were categorized according to themes. Responses from a sub-group of non-infectious diseases/microbiology clinicians were analyzed separately. Statistical analysis was largely descriptive. Categorical data were analyzed using the chi-square test or Fisher’s exact test, as appropriate.

Survey 1 for laboratories sought to determine whether responding laboratories: (i) were aware of changes to fungal nomenclature; (ii) viewed it as appropriate to implement nomenclature changes in reporting practices; (iii) had implemented nomenclature changes in reporting; and (iv) knew of resources available to check currently accepted nomenclature.

Survey 2 for clinicians sought to determine whether the responding clinician: (i) was aware of recent updates to fungal nomenclature; (ii) considered it appropriate for laboratories to include new fungal species names on laboratory reports; (iii) had ever received a laboratory report containing an updated fungal species name; (iv) would consider as clinically significant fungal species reported using only the updated name versus including both the updated and previous name in the report; (v) would utilize available resources to check unfamiliar species names and current nomenclature; and (vi) had further comments or concerns regarding fungal nomenclature.
