# Supplementary material for: Attitudes of Australasian Clinicians and Laboratory Staff to Changing Fungal Nomenclature: Has Mycological Correctness Really Gone Mad?
Source: Microbiol Spectr. 2022 Feb 9;10(1):e02377-21. doi: 10.1128/spectrum.02377-21 (PMC8826822; doi:10.1128/spectrum.02377-21)
Supplement: SUPPLEMENTAL FILE 1 — Supplemental material. Download SPECTRUM02377-21_Supp_1_seq1.pdf, PDF file, 0.4 MB [file spectrum02377-21_supp_1_seq1.pdf]

Survey 1: Laboratory Attitudes to Fungal Nomenclature Changes

All questions are compulsory

We recommend this survey is completed by a laboratory scientist or technical officer who is familiar with the procedures for reporting fungal organisms.

This survey takes approximately 5 minutes.

1. Which of the following best describes your role in the laboratory?

- ☐ Medical scientist or technical officer working at the bench
- ☐ Laboratory manager
- ☐ Quality manager/officer
- ☐ Clinical microbiologist/pathologist
- ☐ Other (please specify)

2. Which of the following best describes your laboratory?

- ☐ Accredited private pathology laboratory
- ☐ Government/Public pathology laboratory
- ☐ Other (please specify)

3. Which of the following best describes your laboratory's mycology capabilities?

- ☐ Bacteriology laboratory with no specific mycology services (most fungal lab requests are referred)
- ☐ Bacteriology laboratory with some limited mycology services (i.e. microscopy, culture, and basic identification)
- ☐ A specialised mycology service or reference laboratory (i.e. microscopy, culture, complex identifications, susceptibility testing)

4. Are you aware that there have been changes to fungal species names in recent years?

- ☐ Yes
- ☐ No

5. Do you think that it is appropriate for clinical mycology laboratories to change organism reporting practices in line with new scientific/taxonomic evidence?

- ☐ Yes
- ☐ No
- ☐ Not sure
- ☐ Yes, for some fungi but not for others (please give details)

6. Has your laboratory changed how it reports some fungal species in response to information about new species names?

- Yes
- No
- Not sure
- Other (please specify)

7. Does your laboratory routinely report fungal organisms using the nomenclature recommended in the RCPAQAP "List of QAP Fungi"?

- Yes
- No
- Not sure
- Other (please specify)

8. If a yeast isolated from a blood culture was identified by MALDI-ToF (or similar IVD identification system) as "*Candida krusei*" how would your laboratory report it?

- "Growth of *Candida krusei*"
- "Growth of *Pichia kudriavzevii*",
- "Growth of *Pichia kudriavzevii* (formerly *Candida krusei*)"
- Not sure
- Other (please specify)

9. If you were unsure of the currently accepted species name for a fungal organism, how would you find out? (Check all that apply)

- Refer to RCPAQAP "List of QAP Fungi"
- Ask a senior scientist or clinical microbiologist
- Perform a 'Google' search
- Perform a search of the scientific literature
- Look up an online database of fungal nomenclature (e.g. Mycobank.org, IndexFungorum.org)
- None of these. Just report the species name as given by the identification platform/laboratory textbooks
- Don't know
- Other (please give details)

Survey 2: Clinician Attitudes to Fungal Nomenclature Changes

All questions are compulsory

This survey should be completed only by clinicians who treat patients with fungal infection and/or interpret fungal culture results from laboratory reports.

This survey takes approximately 5 minutes.

1. Which of the following best describes your area of specialty?

- ☐ Dermatology
- ☐ General Practice
- ☐ Haematology/Oncology
- ☐ ICU
- ☐ Infectious Diseases/Microbiology
- ☐ Internal medicine
- ☐ Paediatrics/Neonatology
- ☐ Renal medicine
- ☐ Respiratory medicine
- ☐ Surgery
- ☐ Other (please specify)

2. Are you aware that there have been scientifically supported changes to fungal species names in recent years?

- ☐ Yes
- ☐ No
- ☐ Not sure

3. In your opinion, how should laboratories report a significant fungal species that has undergone a name change?

- ☐ Report new/updated species names
- ☐ Continue reporting the previous species names that are familiar to clinicians
- ☐ Report new/updated species name **AND** the previous species name, e.g. "Growth of Y, formerly known as X"
- ☐ Other (please specify)

4. Have you ever received a laboratory report for fungal studies that included new/updated species names?

- ☐ Yes
- ☐ No
- ☐ Not sure

5. If you received the following laboratory report of growth from an abscess swab, would you consider it a significant organism?

"Growth of *Candida krusei*"

- ☐ Yes
- ☐ No
- ☐ Not sure

6. If you received the following laboratory report of growth from an abscess swab, would you consider it a significant organism? **"Growth of *Pichia kudriavzeveii*"**

- ☐ Yes
- ☐ No
- ☐ Not sure

7. If you received the following laboratory report of growth from an abscess swab, would you consider it a significant organism? **"Growth of *Nakaseomyces glabrata* (formerly known as *Candida glabrata*)"**

- ☐ Yes
- ☐ No
- ☐ Not sure

8. If you received a laboratory report with a fungal species name that was unfamiliar to you, what steps would you take (tick all that apply)?

- ☐ Take no action
- ☐ Discuss with laboratory and/or pathologist
- ☐ Discuss with Infectious Diseases team
- ☐ 'Google' the reported fungus
- ☐ Search for published studies/case reports relating to the reported fungus
- ☐ Ask a group of experts (e.g. via Ozbug mailing list)
- ☐ Search a database of current fungal species names (e.g. mycobank.org)
- ☐ Other (please specify)

9. In your experience of laboratory reports using new/updated fungal nomenclature, do you have any comments or concerns?

- ☐ No comments
- ☐ Yes (please give details)
